# Smad4 Regulates Ureteral Smooth Muscle Cell Differentiation during Mouse Embryogenesis

**DOI:** 10.1371/journal.pone.0104503

**Published:** 2014-08-15

**Authors:** Jianyun Yan, Lu Zhang, Jinshu Xu, Nishat Sultana, Jun Hu, Xiaoqiang Cai, Jun Li, Pin-Xian Xu, Chen-Leng Cai

**Affiliations:** 1 Department of Developmental and Regenerative Biology, The Mindich Child Health and Development Institute, and The Black Family Stem Cell Institute, Icahn School of Medicine at Mount Sinai, New York, New York, United States of America; 2 Department of Genetics and Genomic Sciences, Icahn School of Medicine at Mount Sinai, New York, New York, United States of America; 3 Department of Histology and Embryology, Southern Medical University, Guangzhou, China; University of Houston, United States of America

## Abstract

Proper formation of ureteral smooth muscle cells (SMCs) during embryogenesis is essential for ureter peristalsis that propels urine from the kidney to the bladder in mammals. Currently the molecular factors that regulate differentiation of ureteral mesenchymal cells into SMCs are incompletely understood. A recent study has reported that Smad4 deficiency reduces the number of ureteral SMCs. However, its precise role in the ureteral smooth muscle development remains largely unknown. Here, we used *Tbx18:Cre* knock-in mouse line to delete Smad4 to examine its requirement in the development of ureteral mesenchyme and SMC differentiation. We found that mice with specific deletion of *Smad4* in *Tbx18*-expressing ureteral mesenchyme exhibited hydroureter and hydronephrosis at embryonic day (E) 16.5, and the mutant mesenchymal cells failed to differentiate into SMCs with increased apoptosis and decreased proliferation. Molecular markers for SMCs including alpha smooth muscle actin (α-SMA) and smooth muscle myosin heavy chain (SM-MHC) were absent in the mutant ureters. Moreover, disruption of *Smad4* significantly reduced the expression of genes, including *Sox9*, *Tbx18* and *Myocardin* associated with SMC differentiation. These findings suggest that Smad4 is essential for initiating the SMC differentiation program during ureter development.

## Introduction

Congenital malformations of the urinary tract resulted from embryonic ureter obstruction lead to hydroureter and/or hydronephrosis with dilated ureter and/or renal pelvis, and are one of the main causations of renal failure among children and young adults [1]–[3]. Currently the molecular mechanisms underlying these congenital defects are poorly understood.

The urinary tract is composed of three important cell types: the inner epithelial cells (urothelium), the outer smooth muscle cells (SMCs) that provide contractility to evacuate urine from kidney to bladder [4], and fibroblasts. During ureter development (from E14.5 in mice), the SMCs start to differentiate from mesenchymal cells surrounding ureter pelvis [5], [6]. Failure in differentiation of ureteral mesenchymal cells into SMCs results in obstruction and hydronephrosis with atrophy of kidney parenchyma [2], [7], [8]. Therefore, it is important to understand molecular factors regulating ureteral SMC differentiation during embryonic development.

Molecular regulation of ureteral SMC differentiation is extremely complex. Previous studies revealed several genes, including *Tbx18*, *Six1*, *Sox9*, *Tszh3* and *β-catenin*, are essential for SMC differentiation during ureter development [2], [7]–[11]. TGF-β super family signals play vital roles in ureter development [10], [12], [13]. In mice, Bmp4 promotes ureter growth and elongation [10], [12]. Decreased Bmp4 signaling results in loss of ureteral smooth muscle formation [14]. As a central mediator in TGF-β signaling [15], Smad4 has been found crucial for vascular SMC differentiation and proliferation [16]. Deletion of *Smad4* in the ureteral mesenchyme leads to a reduced number of SMCs [17]. However, whether Smad4 is essential for ureteral SMC differentiation remains unclear. In particular, the downstream genes through which Smad4 regulates ureter development are largely unknown. In this study, we generated *Tbx18:Cre* knock-in mice to ablate *Smad4* in the ureteral mesenchyme. Our data revealed that Smad4 acts as upstream of several key genes associated with ureteral SMC differentiation, and plays critical roles for ureter development during mouse embryogenesis.

## Materials and Methods

### Animals

*Smad4:floxed* (denoted as *Smad4^f/f^*), *Rosa26:tdTomato* (denoted as *Rosa26^tdTomato^*), and *Tbx18:nlacZ* (denoted as *Tbx18^nlacZ/+^*) mice were described previously [18]–[20]. A new *Tbx18:Cre* (denoted as *Tbx18^Cre/+^*) mouse model was generated by inserting a *Cre-polyA- FRT-Neo-FRT* cassette into the start codon of *Tbx18* locus, with disruption of endogenous ATG. Long range PCR was applied to screen targeted ES cells with 5′ primer P1: 5′-GTGTCCCTGAGTTCAGCTGACTGC-3′ and 3′ primer P2: 5′-CCGGTTATTCAACTTGCACCATGC-3′ (Fig. S1 A). Fragment amplified from the positive ES cells was further confirmed by DNA sequencing. *Tbx18^Cre-FRT-Neo-FRT/+^* mice generated from positive ES cells were crossed to *Flippase* mice [21] to produce *Tbx18^Cre/+^* animals (*Neo* cassette is removed) (Fig. S1). *Smad4^f/f^* mice were bred with *Tbx18^Cre/+^* mice to generate *Tbx18^Cre/+^*; *Smad4^f/+^* doubly heterozygous mice. Mutant *Tbx18^Cre/+^*; *Smad4^f/f^* mice were obtained by mating *Tbx18^Cre/+^*; *Smad4^f/+^* with *Smad4^f/f^* mice. Genomic DNA was prepared from yolk sacs or tail biopsies for genotyping. Cervical dislocation and carbon dioxide inhalation were applied to euthanize mice. Mouse husbandry was carried out according to an approved IACUC protocol at the Icahn School of Medicine at Mount Sinai (Permit LA09-00494), and is in compliance with institutional and governmental regulation (PHS Animal Welfare Assurance A3111-01).

### X-gal staining

Ureters from *Tbx18^nlaZ/+^* mouse were checked for β-galactosidase activity by X-gal staining. Mouse ureters were treated with fixation solution (4% paraformaldehyde in PBS) for 30 min at 4°C. The fixed ureters were washed twice with PBS and then stained with X-gal solution (5 mM Potassium Ferricyanide, 5 mM Potassium Ferrocyanide, 2 mM MgCl_2_, 1 mg/ml X-gal) for 12 hours at room temperature. Tissues were visualized with a Leica steromicroscope.

### Histology

Mouse ureters were washed in PBS and fixed with 4% paraformaldehyde overnight at 4°C, dehydrated in an ascending ethanol series (25%, 50%, 75%, 100%) followed by two changes of 100% xylene. The tissues were then immersed in liquid paraffin for 2 hours and left on a cold plate until wax was solidified. Paraffin blocks were cut into 6 µm in thickness on a microtome. The sections were stained with Hematoxylin and Eosin (H&E) using standard procedures.

### Immunofluorescence and RNA *in situ* hybridization

Mouse ureters were fixed in 4% paraformaldehyde for 30 min and embedded in Optimal Cutting Temperature compound (Tissue-Tek). Frozen samples for immunohistochemistry were cut into 6 µm in thickness. Primary antibodies used in this study were as follows: rabbit anti-Smad4 (1∶100, Millipore), rabbit anti-Sox9 (1∶300, Millipore), mouse anti-αSMA (1∶100, Sigma), rabbit anti-SM-MHC (1∶100, Biomedical Technologies), rabbit anti-Uroplakin (1∶100, a generous gift from Dr. Tung-Tien Sun, NYU) [22]. Alexa Flour 488 or 594 conjugated secondary antibodies (1∶500; Invitrogen) were applied to detect the corresponding primary antibodies. Section RNA *in situ* hybridization was carried out on 12-µm cryosections with methods described previously [23], [24].

### Proliferation and apoptosis analysis

For cell proliferation assay, pregnant mice were intraperitoneally injected with 10 mM EdU (Invitrogen) in PBS (5 mg per 100 g body weight). Embryos were harvested 4 hours later and fixed in 4% paraformaldehyde for 30 min at 4°C, and were embedded in OCT compound. Cell proliferation was assessed on 6 µm frozen sections using Click-iT EdU Cell Proliferation Assay Kit (Invitrogen). To quantify cell proliferation, 8 ureter sections were prepared from each embryo, and sections are from comparable locations in the control and mutant. Two embryos of each genotype were analyzed. Proliferation index was calculated as the percentage of EdU positive cells relative to ureteral mesenchymal cells and ureteric epithelial cells, respectively. Apoptosis assay was performed on 6 µm frozen sections using in situ Cell Death Detection Kit (Roche) according to the manufacturer's instructions. Nine representative ureter sections from each of three embryos with control or mutant genotype at comparable locations were used for apoptosis assay. Apoptosis index was calculated as percentage of TUNEL positive cells relative to ureteral mesenchymal cells.

### Quantitative real-time PCR

Total RNA was isolated from ureters with Trizol reagent (Invitrogen) according to the manufacturer's instructions. QuantiTect Reverse Transcription Kit (Qiagen) was used to synthesize First-strand cDNA from total RNA. Quantitative PCR was performed using StepOnePlus PCR system and SYBR green detector (Qiagen). All transcripts were normalized to *β-actin*. The relative amounts of mRNA were calculated using the comparative Ct (threshold cycle) method. Primers used in this study are listed in Table 1. Data are presented as mean ± SD. Statistical analysis was performed by *t*-test and a value of *p*<0.05 was considered significant.

**Table 1:** Primer sequences for quantitative PCR.

Gene	Primer sequence (5′-3′)
Myocd	CATTCGCCTTTGAGGATGAC
	CTGAGCCAGGAGTGAGATCC
Acta2	GAGGCACCACTGAACCCTAA
	CATCTCCAGAGTCCAGCACA
Myh11	AACACAGACCAGGCATCCAT
	CTTTGGTCTGAGCCTTCTGC
Mycn	AGCACCTCCGGAGAGGATAC
	CGCACAGTGATCGTGAAAGT
Sox9	TGCAGCACAAGAAAGACCAC
	CCCTCTCGCTTCAGATCAAC
Tbx18	TGGGGAGACTTGGATGAGAC
	GGACAGATCATCTCCGCAAT
Tshz3	AAGCATCATGCCGAGGAG
	CTCCATCTGCCGCTTGTT
Ptch1	TACGTGGAGGTGGTTCATCA
	AGGCATAGGCAAGCATCAGT
β-actin	TGTTACCAACTGGGACGACA
	GGGGTGTTGAAGGTCTCAAA

## Results

### *Tbx18^Cre/+^* mice mediate specific recombination in the ureteral mesenchyme and SMCs

**Figure 1:**
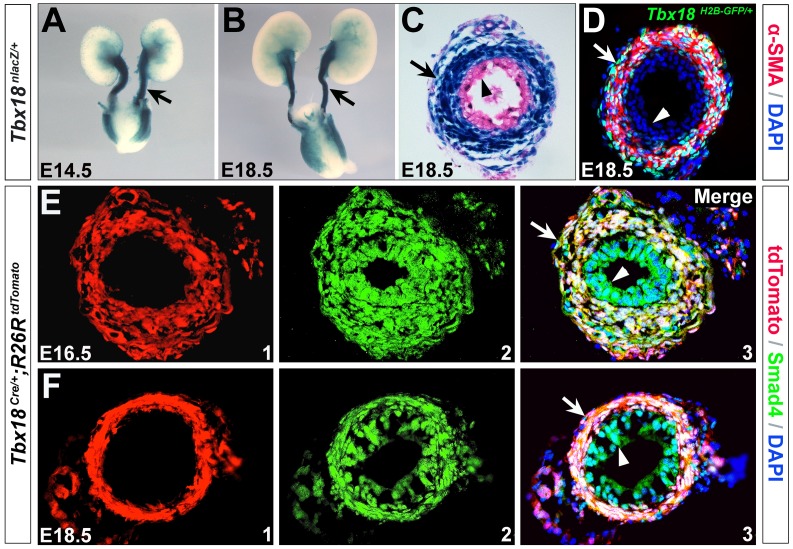
Expression and lineage analysis of Tbx18 in the developing ureters. (A-C) *Tbx18* expression in the developing ureters. A and B are whole-mount X-gal staining of *Tbx18^nlacZ/+^* urinary system at E14.5 and E18.5, respectively. C is a transverse section of the ureter tube at E18.5. (D) Immunostaining of α-SMA (red) on *Tbx18^H2BGFP/+^* ureter (transverse section) at E18.5. (E,F) Immunofluorescence analysis of Smad4 with *Tbx18* lineage on *Tbx18^Cre/+^;Rosa26^tdTomato^* ureter at E16.5 and E18.5. Arrowheads indicate urothelium and arrows indicate ureter tube (A and B) or ureteral SMCs (C-F).

Previous studies showed that nuclear *lacZ* (*nlacZ*) expression in the *Tbx18^nlacZ/+^* mice, and *H2B-GFP* expression in the *Tbx18^H2B-GFP/+^* mice faithfully recapitulated endogenous *Tbx18* expression in various organs, including the heart, limb, somite and hair follicle during embryogenesis in mice [19], [25], [26]. We utilized *Tbx18^nlacZ/+^* and *Tbx18^H2B-GFP/+^* mice to examine *Tbx18* expression in the developing ureter during mouse embryogenesis. X-gal staining revealed that *Tbx18* is highly expressed in the ureter tube at E14.5-18.5 (Fig. 1A-C). Immunostaining indicated *Tbx18* is confined to the developing ureteral mesenchyme and SMCs (Fig. 1D). Given the specific expression of *Tbx18* during embryogenesis, we generated *Tbx18^Cre-FRTNeoFRT/+^* knock-in mice by inserting a *Cre-polyA-FRT-Neo-FRT* cassette into the start codon of *Tbx18* locus (Fig. S1). *Tbx18^Cre/+^* mice were obtained by removing the *Neo* cassette through crossing *Tbx18^Cre-FRTNeoFRT/+^* mice to *Flippase* mice [21] (Fig. S1). Subsequently, we determined *Tbx18* cell fates by crossing *Tbx18^Cre/+^* mice to *Rosa26^tdTomato^* reporter line [20]. Lineage analysis of *Tbx18^Cre/+^*; *Rosa26^tdTomato/+^* doubly heterozygous animals revealed that *Tbx18* progeny encompass ureteral mesenchyme and its corresponding smooth muscles, but not epithelial cells (urothelium) (Fig. 1E,F).

### Disruption of Smad4 in the ureteral mesenchyme causes hydroureter and hydronephrosis

To investigate the potential role of Smad4 in ureter development, we first performed immunostaining and found that Smad4 is universally expressed in both ureteral mesenchyme and epithelial cells, and is co-expressed with *Tbx18* lineages in the mesenchyme and SMCs (Fig. 1E,F, and data not shown for E13.5-E15.5). We examined *Tbx18^Cre/+^* ureters found they developed normally without defects at birth (P0) when compared to their wild type littermates (Fig. S2A,B). Further examination of *Tbx18* mRNA expression in the ureters by quantitative RT-PCR (qRT-PCR) showed no significant difference between the wild type and *Tbx18^Cre/+^* mice at this stage (Fig. S2C).

**Figure 2:**
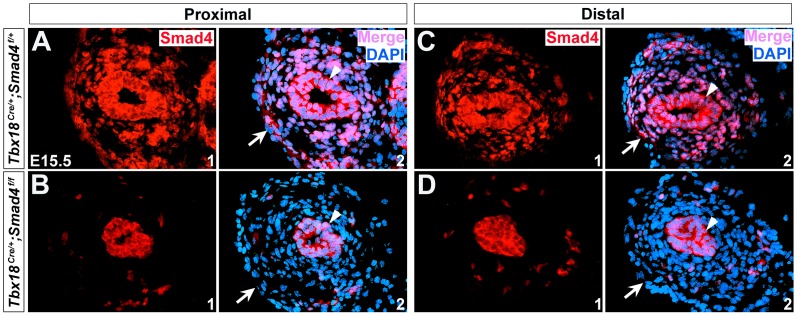
Inactivation of Smad4 in *Smad4^CKO^* ureter. (A-D) Immunostaining of Smad4 in the control (A and C, *Tbx18^Cre/+^;Smad4^f/+^*) and mutant ureters (B and D, *Smad4^CKO^*) in the proximal and distal positions at E15.5. Arrowheads indicate urothelium and arrows indicate ureteral mesenchyme and SMCs.

**Figure 3:**
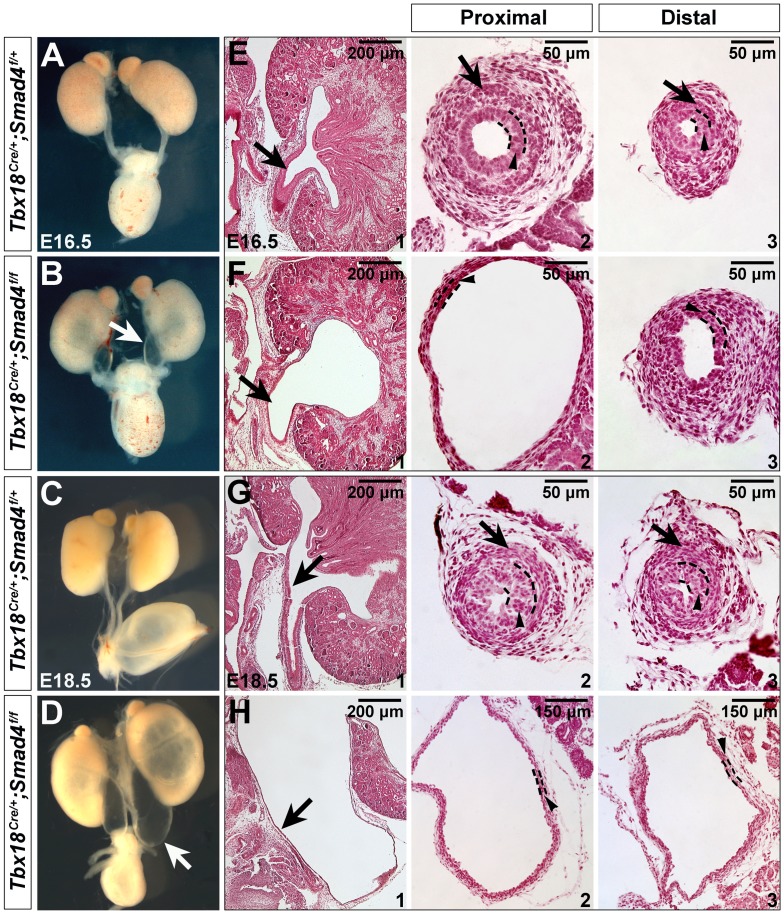
Disruption of Smad4 leads to hydroureter and hydronephrosis. (A-D) Morphology of urinary system in the control and mutant mice at E16.5 (A and B) and E18.5 (C and D). (E-H) Hematoxylin and Eosin staining of kidney sagittal sections (E1/F1/G1/H1), and transverse sections of ureter tube in the proximal (E2/F2/G2/H2) and distal positions (E3/F3/G3/H3) at E16.5 and E18.5. Arrows in B/D and E1/F1/G1/H1 indicate ureter tube, and in E2/F2/G2/H2 and E3/F3/G3/H3 indicate ureteral SMCs. Arrowheads indicate urothelium.

We crossed *Tbx18^Cre/+^;Smad4^f/+^* double heterozygous mice to *Smad4^f/f^* mice and *Tbx18^Cre/+^*; *Smad4^f/f^* mutant animals (denoted as *Smad4^CKO^*) were collected for analysis. Littermates with *Tbx18^Cre/+^;Smad4^f/+^* genotype were utilized as controls. To determine if *Tbx18^Cre/+^* mediates efficient recombination in the ureteral mesenchyme and SMCs, we performed immunostaining in the mutant tissues. It showed that Smad4 expression was absent in the ureteral mesenchyme and smooth muscles (notched arrows in Fig. 2, data not shown for E14.5), but was unaffected in ureteric epithelium (arrowheads in Fig. 2A-D), suggesting that Smad4 was specifically disrupted in ureteral mesenchyme and SMCs on *Smad4^CKO^* embryos. At E16.5, *Smad4^CKO^* embryos displayed a prominent bilateral hydroureter and hydronephrosis (Fig. 3A, B), and the defects became more severe at E18.5 (Fig. 3C,D). Histological analysis of these embryos showed dilated and thin-walled ureters with renal parenchymal atrophy and dilation of the renal pelvis (Fig. 3E-H). The mutant mice cannot survive beyond 24 hours after birth, whereas control mice with *Tbx18^Cre/+^*; *Smad4^f/+^* genotype develop normally and can survive to adulthood without any apparent morphogenetic defects.

### Smad4 is essential for ureteral mesenchymal cell differentiation

**Figure 4:**
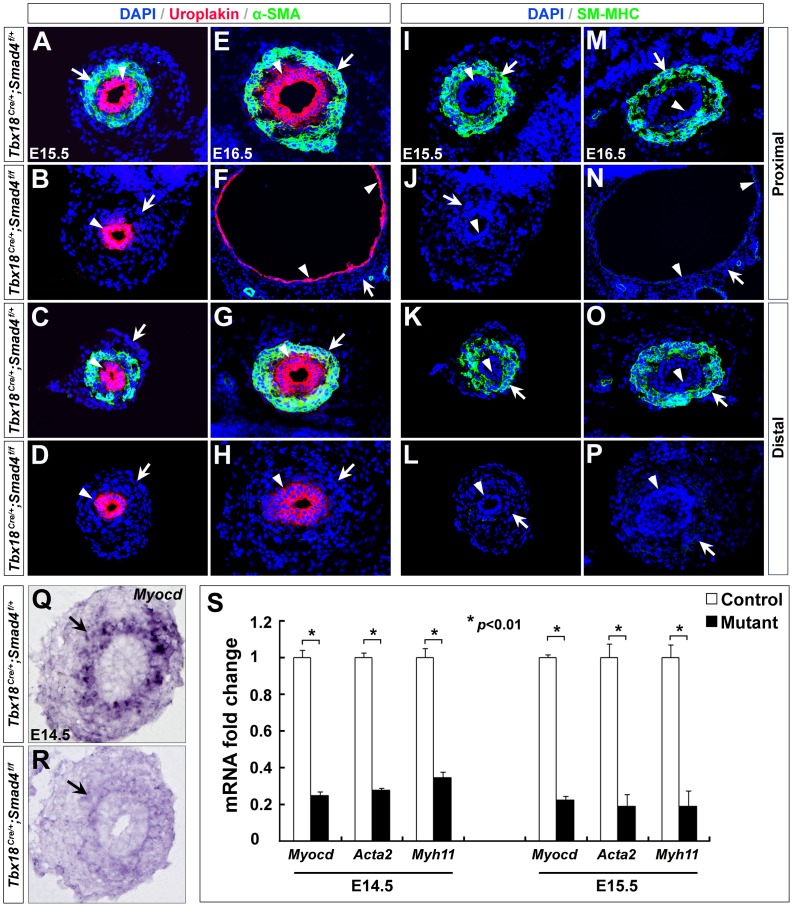
SMC differentiation is perturbed in *Smad4^CKO^* ureters. (A-H) Immunostaining of α-SMA and Uroplakin in the control (A/C/E/G) and mutant ureters (B/D/F/H) at E15.5 and E16.5. Arrowheads indicate Uroplakin (urothelium) and arrows indicate α-SMA staining (SMCs). (I-P) Immunostaining of SM-MHC in the control (I/K/M/O) and mutant embryos (J/L/N/P) at E15.5 and E16.5. Arrowheads indicate urothelium and arrows indicate SMCs. (Q,R) RNA *in situ* hybridization revealed *Myocd* expression is downregulated in the mutants (arrows) at E14.5. (S) Relative expression of *Myocd*, *Acta2* and *Myh11* was determined by qRT-PCR in the mutant and control littermate ureters at E14.5 and E15.5.

SMCs are essential for ureter peristalsis that propels urine towards bladder. Previous studies suggested that hydronephrosis and hydroureter could primarily result from malformed SMCs [2], [7], [8]. To determine whether the hydronephrosis and hydroureter defects in *Smad4^CKO^* embryos were due to insufficient differentiation of the ureteral SMCs, we examined mutant ureters with SMC markers. During embryonic development, ureteral SMC differentiation is characterized by increased expression of contractile SMC markers, such as α-SMA and SM-MHC [27], from E14.5 to E16.5. While we found α-SMA and SM-MHC were highly expressed in the ureteral SMCs at E15.5 and E16.5 in the controls, they could be barely detected in the mutant embryos (notched arrows in Fig. 4A-P). Similar observations were made for *Myocardin* (*Myocd*), a key regulator and marker of SMC differentiation, on E14.5 mutant ureters (notched arrows in Fig. 4Q,R). Further qRT-PCR showed that *Myocd*, *Acta2* and *Myh11* were down-regulated in the *Smad4^CKO^* ureters by 75%, 72% and 65% at E14.5, and by 78%, 81% and 81% at E15.5, respectively (Fig. 4S, *p*<0.01). Moreover, we investigated whether *Smad4* deficiency in the ureteral mesenchyme also affects urothelium formation. Uroplakin, a marker for urothelium, was still expressed in the mutant embryos, suggesting that urothelial development was unaffected in *Smad4^CKO^* mice (arrowheads in Fig. 4A-H).

### Increased apoptosis and decreased proliferation in *Smad4^CKO^* ureteral mesenchyme

**Figure 5:**
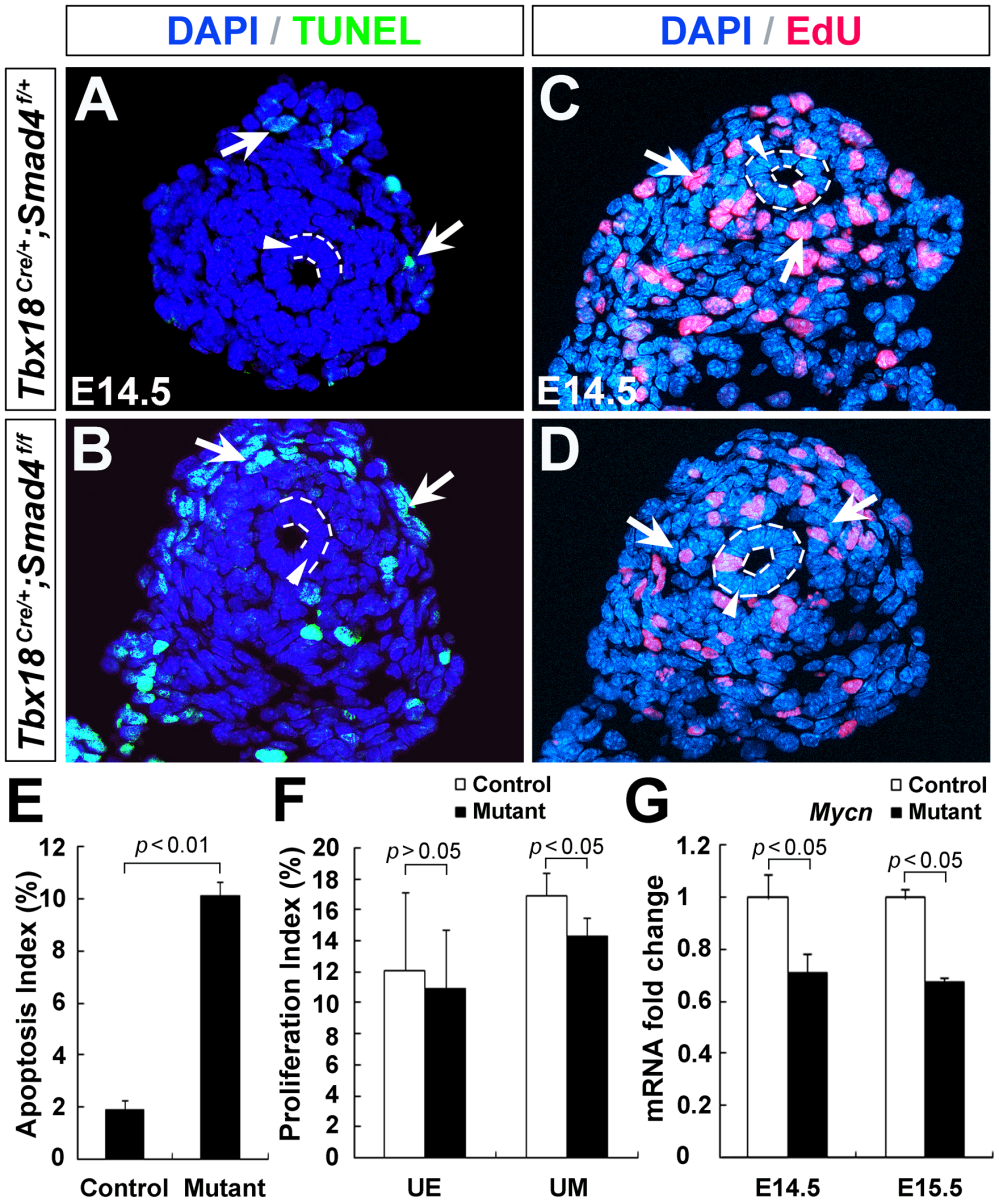
Apoptosis and cell proliferation in *Smad4^CKO^* ureters. (A, B) Apoptosis was assessed by TUNEL. Arrows indicate TUNEL positive cells. (C, D) Cell proliferation was analyzed by EdU labeling. Arrows indicate proliferating cells. (E) Statistical analysis of TUNEL positive cells. (F) EdU positive cells were quantified. (G) Relative *Mycn* mRNA expression was measured by qRT-PCR.

Cell apoptosis and proliferation are highly associated with hydroureter during ureter development [2], [5], [28], [29]. We examined the effect of *Smad4* deletion on cell apoptosis and proliferation in the ureteral mesenchyme. TUNEL analysis revealed no change of apoptosis in ureteric epithelium between control and mutant at E14.5. In contrast, we found a increased number of apoptotic cells in the mutant ureteral mesenchyme (Fig. 5A,B,E, control: 1.88±0.38%; mutant: 10.1±0.58%; *p*<0.01). Moreover, we assessed mesenchymal cell proliferation by EdU incorporation. The ureteral mesenchymal cell proliferation rate was ∼15% less in the *Smad4^CKO^* mutants at E14.5 (control: 16.93±1.44%; mutant: 14.32±1.22%; *p*<0.05), yet cell proliferation rate in the epithelium was unaffected (Fig. 5C,D,F, *p*>0.05). Consistent with these observations, expression of *Mycn*, a pro-proliferative factor, was significantly reduced in *Smad4^CKO^* ureters at E14.5 and E15.5 (Fig. 5G, *p*<0.05).

### *Sox9* and *Tbx18* expression is down-regulated in *Smad4^CKO^* ureter

**Figure 6:**
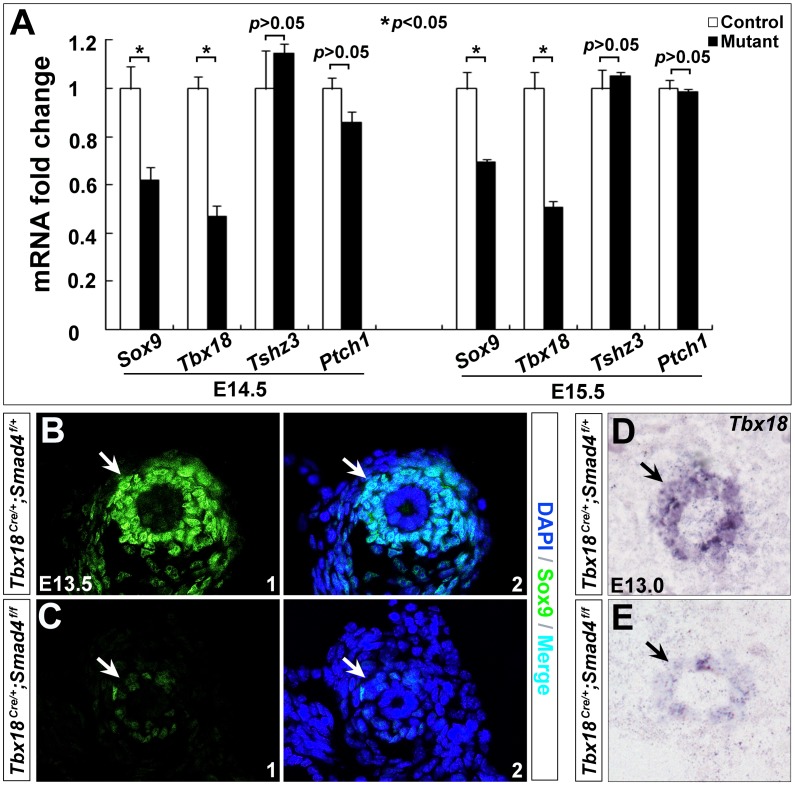
Reduced *Sox9* and *Tbx18* expression in *Smad4^CKO^* ureters. (A) qRT-PCR was performed to determine expression of *Sox9*, *Tbx18*, *Tshz3* and *Ptch1*. (B,C) Immunostaining of Sox9 in control (B) and mutant embryos (C) at E13.5. (D,E) RNA *in situ* hybridization of *Tbx18* in the control (D) and mutant embryos (E) at E13.0, respectively. Arrows indicate positive staining of cells in the controls with corresponding regions in the mutants.

We next performed qRT-PCR to identify genes as potential downstream of Smad4 signaling for ureteral SMC differentiation. Given that *Sox9*, *Tbx18*, *Tszh3* and *Shh* have been shown important in regulating ureteral SMC differentiation [2], [5], [7], [8], we assessed the effect of *Smad4* deficiency on these genes during mouse embryonic development. qRT-PCR revealed ∼38% reduction in *Sox9* and ∼53% reduction in *Tbx18* on the mutant ureters at E14.5, and ∼31% reduction in *Sox9* and ∼50% reduction in *Tbx18* at E15.5 (*p*<0.05), yet *Tszh3* and *Ptch1* (receptor of *Shh*) expression was not significantly changed (Fig. 6A, *p*>0.05). Further immunostaining and RNA *in situ* hybridization showed that Sox9 was down-regulated in the mutant ureteral mesenchyme as early as E13.5 (Fig. 6B,C), and *Tbx18* mRNA was significantly decreased in the mutant mesenchyme at E13.0 (Fig. 6D,E). These data suggest that *Sox9* and *Tbx18* may act as downstream of Smad4 to govern ureteral smooth muscle differentiation during mouse embryogenesis.

## Discussion

Although a few genes (e.g., *Tbx18*, *Sox9*, *Tszh3* and *β-catenin*) have been identified crucial for ureter development [2], [7]–[9], the interaction network of these genes with other factors in regulating ureteral SMC development remains to be determined. In this study, we found loss of Smad4 in the ureteral mesenchyme led to failure of SMC differentiation, resulting in severe hydroureter and hydronephrosis during mouse embryogenesis. Smad4 acts as upstream of *Sox9*, *Tbx18* and *Myocardin*, and is required for their normal expressions in ureter development [2], [8].

### *Tbx18^Cre/+^* mouse model as a robust genetic tool to study ureteral mesenchyme and SMC development

The expression analysis of *Tbx18* with *Tbx18^nlacZ/+^* and *Tbx18^H2B-GFP/+^* mice revealed that *Tbx18* was specifically expressed in the ureteral mesenchyme and SMCs (Fig.1A-D), consistent with the RNA *in situ* hybridization[2]. Lineage tracing experiments with *Tbx18^Cre/+^*; *Rosa26^tdTomato^* animals revealed *Tbx18* progeny include undifferentiated ureteral mesenchyme and its SMC derivatives, but not ureteric epithelium. Therefore, the *Tbx18^Cre/+^* animal could be utilized as an excellent genetic tool to dissect functions of genes in the developing ureteral mesenchyme and/or SMCs during mouse embryogenesis with Cre-loxP technology. Comparing to *Tbx18^Cre/+^* knock-in mice, *Pax3-Cre* transgenic line introduces recombination in ureteral mesenchyme (including SM layers and the adventitia) and metanephric mesenchyme (including the glomeruli and the proximal and distal tubules) [28]. The BAC transgenic *Tbx18-Cre* line was also generated [30]. However, it may not fully recapitulate endogenous *Tbx18* expression thus it is not clear whether it introduces effective recombination in all the ureteral mesenchymal cells. Evidence in supporting this argument is that*Tbx18-Cre* mouse seems not able to mediate effective recombination in the somites of mouse embryos [30].

### Smad4 is required for ureteral SMC differentiation from mesenchyme

Previous studies demonstrated that Bmp4 plays an important role in ureter development [10], [12], [31]. Smad4 is a central mediator of TGF-β/Bmp signaling pathway [32], [33] and it regulates morphogenesis of various organs during embryonic development [34]–[36]. Our study showed that Smad4 is expressed in both ureteral mesenchyme and epithelium during ureter development. Mice with genetic deletion of *Smad4* in ureteral mesenchyme displayed hydroureter and hydronephrosis as early as E16.5, and the mutant mesenchymal cells failed to differentiate into SMCs at E15.5. *Myocd*, a critical transcription factor required for expression of SMC contractile proteins (αSMA, SM22α and SM-MHC) [37], is dramatically down-regulated in the mutant ureters. Ureter SMC developmental defects are highly associated with defective cell proliferation and survival [9], [10], [28]. In *Smad4^CKO^* embryos, we also detected less proliferative ureteral mesenchymal cells with apoptosis at E14.5. The increased mesenchymal cell death in *Smad4^CKO^* mice may be attributed to the reduced *Myocd* expression, given that *Myocd* is required for cell survival [38]. Furthermore, loss of *Smad4* resulted in down-regulation of *Mycn* in the ureter. Mycn (Nmyc) is a Myc transcription factor and it plays critical roles in cell proliferation [16], [39], [40]. In the developing hearts, *Mycn* is a direct downstream target of Smad4 and inactivation of Smad4 down-regulates *Mycn* expression [39]. *Mycn* may play a key role in Smad4-mediated signaling cascades for the ureteral mesenchymal cell proliferation. Collectively, these observations suggested that *Smad4* signals are crucial for SMC differentiation, proliferation and survival during ureter development.

A recent study has attempted to disrupt *Smad4* with *Tbx18-Cre* transgenic line, and the mutant embryos displayed hydronephrosis at E17.5 [17]. The defects in these animals were not as severe as our *Smad4^CKO^* mice: although the ureteral SMC number was reduced, SMC differentiation still occurred and thickness of smooth muscle layer was not affected. Moreover, no significant difference was found in cell survival and proliferation in the mesenchyme or SMCs. The discrepancies may be due to Cre excision efficiency: *Tbx18-Cre* transgenic line may not able to mediate efficient excision in the ureteral mesenchymal cells [17]. Some of the mesenchymal cells in the mutant ureters may still maintain Smad4 expression and thus develop normally into SMCs. *Tbx18^Cre/+^* knock-in mouse line used in our study can mediate efficient ablation in almost all the ureteral mesenchymal cells (Fig. 2A-D), and therefore SMC differentiation does not occur in *Smad4^CKO^* ureters.

### *Sox9* and *Tbx18* act as downstream of Smad4 during ureteral mesenchymal cell development

Genetic regulation of ureteral SMC differentiation is a complex process. Pathways underlying this process are still largely to be determined. To date, a few genes, including *Sox9* and *Tbx18*, were identified as key regulators for ureteral SMC differentiation [2], [8]. *Sox9* is a transcription factor highly expressed in ureteral mesenchyme at E12.5 and E13.5 [41]–[43]. Deletion of *Sox9* in ureteral mesenchyme results in hydroureter and hydronephrosis [8], mimicking defects in *Smad4^CKO^* embryos (Fig. 3A-D). Mesenchymal cells in *Sox9^−/−^* ureters do not differentiate into SMCs [8]. *Sox9* and *Tshz3* regulate smooth muscle differentiation program by controlling the activity of *Myocd* [44], a key regulator for SMC differentiation [45]–[47]. Previous studies also showed that Smad4 regulates *Sox9* expression in limb and heart development [48], [49]. In *Smad4^CKO^* embryos, we also found *Sox9* expression was down-regulated (Fig. 6A-C). *Tbx18* is a transcription factor highly expressed in ureteral mesenchyme and is essential for SMC differentiation [2], [50]. Loss of *Tbx18* in mice results in defective mesenchymal cell proliferation and SMC differentiation [2], similar to *Smad4^CKO^* embryos. Disruption of *Smad4* down-regulates *Tbx18* expression (Fig. 6A,D,E). Based on these observations, we speculate that *Smad4* may act as upstream of both *Sox9* and *Tbx18*, and that *Smad4*-*Sox9/Tbx18* signaling cascades may regulate ureteral mesenchymal cell development through *Myocd*.

In addition, microRNA-21 (miR-21) has been shown to promote vascular SMC differentiation by inducing expression of SMC contractile proteins including αSMA, calponin and SM22a [51]. MiR-21 also regulates vascular SMC growth and survival by silencing expression of PTEN and increasing expression of BCL2 [52]. TGF-β and Bmps (Bmp4) signaling elevates the expression of mature miR-21 [51], [53]. It is not certain whether miR-21 mediates ureteral SMC differentiation as downstream of Smad4, although miR-21 biogenesis in the vascular SMCs is only regulated by ligand-specific Smad proteins [51].

In the future, it will be of interest to determine whether Smad4 directly activates the expression of *Sox9/Tbx18/Myocd* by binding their regulatory elements, and whether miR-21 is affected by Smad4 during ureteral development. Furthermore, given the critical roles of Smad4 in ureter development, it will be also of interest to perform genetic studies to determine whether Smad4 and its associated downstream genes Sox9, Tbx18, and Myocd are mutated in human patients with congenital urinary tract malformations.

## Supporting Information

Figure S1 **Generation of** ***Tbx18^Cre/+^*** **knock-in mouse.** (A) Schematic representation of targeting strategy. A *Cre-polyA-FRT-Neo-FRT* cassette was introduced into the *Tbx18* genomic locus (6 bp upstream of the ATG). The *Neo* cassette is flanked by two FRT sites. *Tbx18^Cre-FRT-Neo-FRT/+^* mice were generated from the positive ES cells. *Flippase* deleter mice were crossed to *Tbx18^Cre-Neo^* mice to remove *Neo* cassette. (B) Long range PCR analysis of genomic DNA from targeted ES cells. A 4.2-kb fragment was amplified with 5′ primer external to the 5′ arm (P1) and 3′ primer within *Neo* cassette (P2).
(TIF)

Figure S2 **Normal ureter development in** ***Tbx18^Cre/+^*** **mice.** (A,B) Comparisons of the urinary system from *Tbx18^Cre/+^* knock-in mice and their wild type littermates at birth (P0). (C) *Tbx18* mRNA expression in ureters measured by qRT-PCR does not show significant difference between *Tbx18^Cre/+^* and wild type mice. β-actin was used as an internal reference gene.
(JPG)

Checklist S1 **ARRIVE Checklist.**
(DOC)
