# Supplementary figures and images for: Smad4 Regulates Ureteral Smooth Muscle Cell Differentiation during Mouse Embryogenesis
Source: PLoS One. 2014 Aug 15;9(8):e104503. doi: 10.1371/journal.pone.0104503 (PMC4134214; doi:10.1371/journal.pone.0104503)

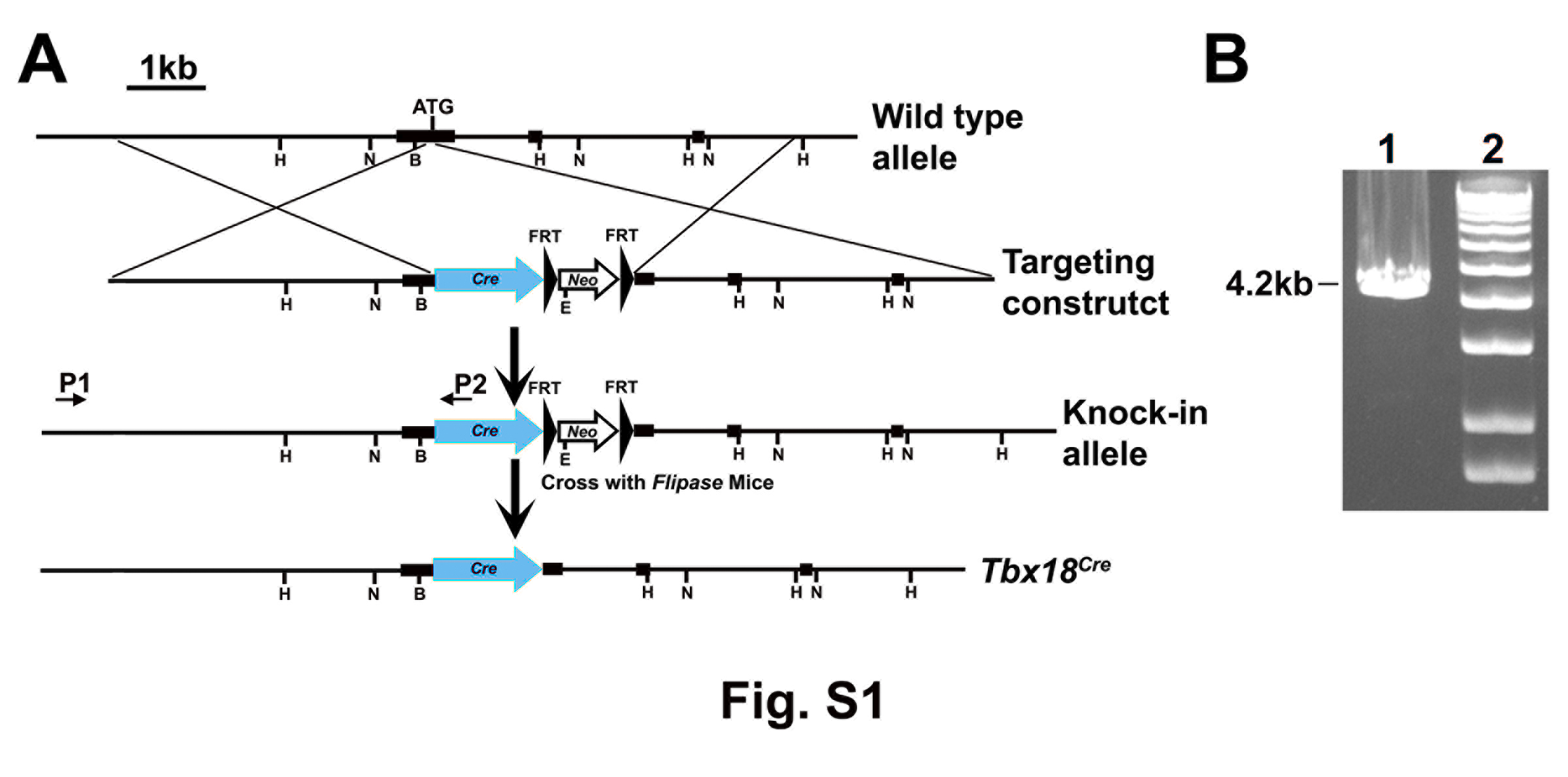

Supplement: Figure S1 — Generation of Tbx18Cre/+ knock-in mouse. (A) Schematic representation of targeting strategy. A Cre-polyA-FRT-Neo-FRT cassette was introduced into the Tbx18 genomic locus (6 bp upstream of the ATG). The Neo cassette is flanked by two FRT sites. Tbx18Cre-FRT-Neo-FRT/+ mice were generated from the positive ES cells. Flippase deleter mice were crossed to Tbx18Cre-Neo mice to remove Neo cassette. (B) Long range PCR analysis of genomic DNA from targeted ES cells. A 4.2-kb fragment was amplified with 5′ primer external to the 5′ arm (P1) and 3′ primer within Neo cassette (P2). (TIF) [file pone.0104503.s001.tif]

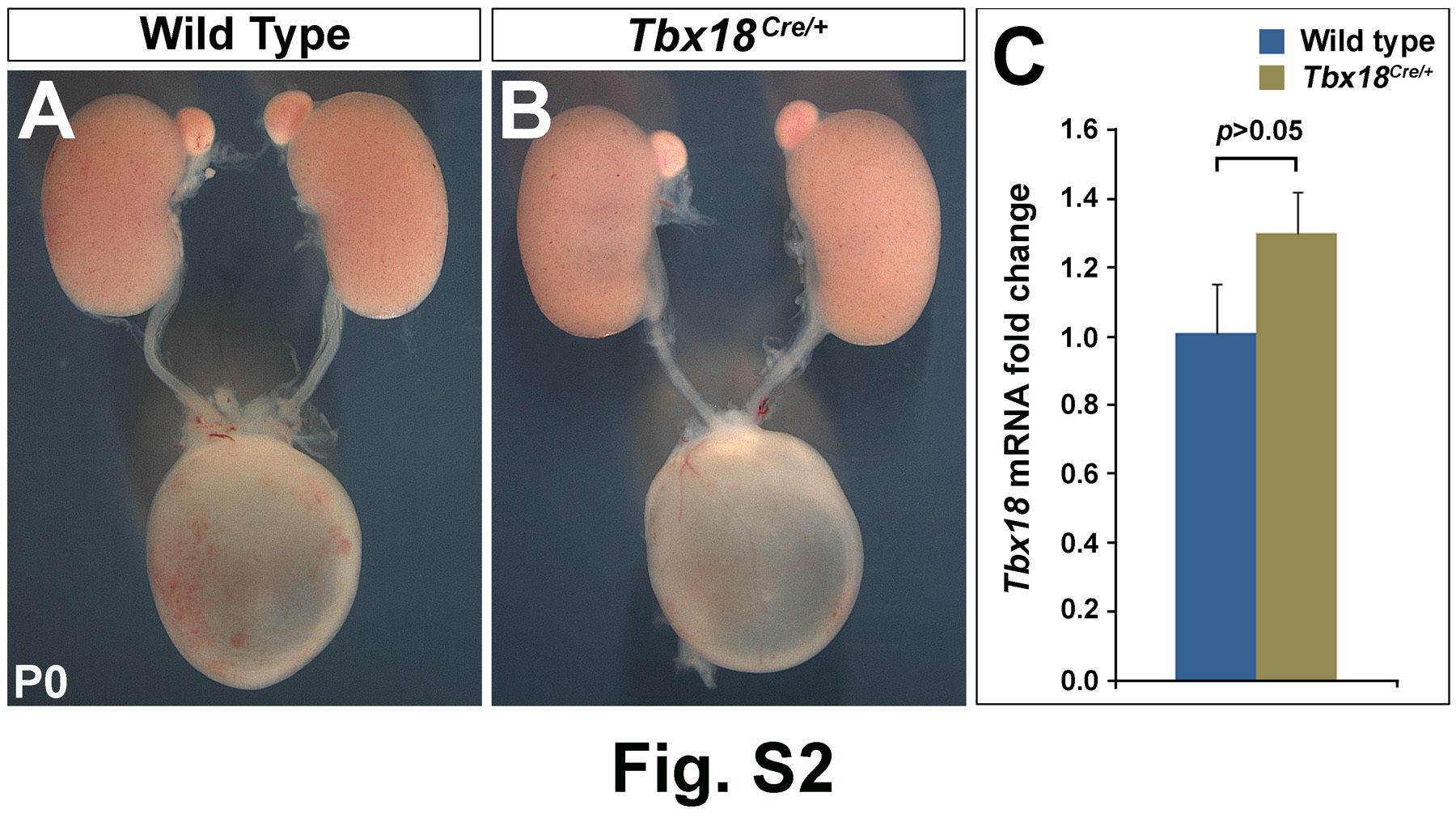

Supplement: Figure S2 — Normal ureter development in Tbx18Cre/+ mice. (A,B) Comparisons of the urinary system from Tbx18Cre/+ knock-in mice and their wild type littermates at birth (P0). (C) Tbx18 mRNA expression in ureters measured by qRT-PCR does not show significant difference between Tbx18Cre/+ and wild type mice. β-actin was used as an internal reference gene. (JPG) [file pone.0104503.s002.jpg]
